# Migration and Transformation of Cd in Pig Manure–Insect Frass (*Hermetia illucens*)–Soil–Maize System

**DOI:** 10.3390/ijerph20010060

**Published:** 2022-12-21

**Authors:** Xiaobo Wang, Nan Wu, Ye Ma, Zhiqiang Wang, Ruijie Cai, Xiaoyan Xu

**Affiliations:** 1 College of Agronomy and Resource and Environment, Tianjin Agricultural University, Tianjin 300392, China; 2 College of Engineering and Technology, Tianjin Agricultural University, Tianjin 300392, China

**Keywords:** black soldier fly, maize, cadmium, larvae frass, cadmium speciation, swine manure

## Abstract

Little is known about the fate of heavy metals in the recycling system of animal manure–black soldier fly larvae (BSFL) transformation-larval frass application. In this work, BSFL-transformed pig manure with different concentrations of exogenous cadmium (Cd) (0, 3, 15, 30 mg kg^−1^), and the obtained BSFL frass fertilizer were further used in pot experiments of maize planting to explore Cd migration during the whole recycling system. Results showed that Cd addition to pig manure had no significant effects on BSFL growth or BSFL transformation performance. The Cd concentrations in BSFL frass were 10.9–19.8% lower than those in pig manure, while those in BSFL bodies were 2.3–4.0-times those of pig manure. For maize planting, only 30 mg kg^−1^ of Cd treatment significantly inhibited maize growth. The BSFL frass application (under exogenous Cd treatment) enhanced Cd contents in the aboveground and underground parts of maize (3.3–57.6-times) and those in soil (0.5–1.7-times) compared with CK (no Cd addition). Additionally, 61.2–73.5% of pig manure-sourced Cd was transformed into BSFL frass and the rest entered BSFL bodies. Only a small part (0.31–1.34%) of manure-sourced Cd entered maize plants. BSFL transformation decreased the proportions of weak acid-dissolved Cd from 44.2–53.0% (manure) to 37.3–46.0% (frass). After frass application, the proportions of weak acid-dissolved Cd in soil were further decreased to 17.8–42.5%, while the residual fractions of Cd increased to 27.2–67.7%. The findings provided a theoretical basis for the rational application of BSFL frass fertilizers sourced from heavy-metal-contaminated manure.

## 1. Introduction

The rapid development of the livestock and poultry breeding industry has produced a large amount of animal manure while meeting human needs [1]. Traditionally, livestock manure after composting can be directly returned to the field to increase soil fertility and improve crop growth [2]. However, various heavy metals or trace elements are often added to feed during the breeding process in order to improve the utilization rate of feed and promote animal growth. Due to the low utilization rate of trace elements by animals, most of them are excreted through animal manure. High levels of various heavy metal elements exceeding the standard are often reported in livestock manure [3,4]. Consequently, there are many problems associated with manure contamination, such as the impacts of manure-sourced heavy metals on soil and groundwater, especially when large amounts of livestock manure are applied as fertilizer in agriculture [5].

Black soldier fly larva (BSFL) (*Hermetia illucens* L.) is a saprophytic insect, possessing the characteristics of rapid reproduction, large biomass, wide food habit, high transformation rate, easy management, and low feeding cost. More importantly, BSFL can reduce the contents of pathogens and antibiotics in manure during the transformation process, so it has been widely used to treat livestock manure in recent years [6,7]. Meanwhile, the transformed products of BSFL (e.g., larval protein and larval frass) have high utilization value [8,9,10], providing an economical and feasible means of manure treatment [11,12].

Previous studies have shown that BSFL have strong tolerance to heavy metals in diverse organic waste [13,14,15]. However, heavy metals derived from animal manure will remain in the bodies and frass of BSFL after manure transformation, which brings risks to the further utilization of larval biomass and frass. Cadmium (Cd), in particular, a common metal existing in animal manure, can be highly accumulative in BSFL bodies [14,16,17] and, thus, merits considerable attention. On the one hand, when using BSFL bodies as animal feed, excessive Cd in BSFL bodies would cause Cd poisoning of animals and reduce production performance. On the other hand, when using BSFL frass as organic fertilizer in the soil, excessive Cd in soil would inhibit the normal growth of plants.

Few studies have reported the fate of heavy metals in the recycling system of animal manure–BSFL transformation-larval frass application or the impacts of heavy metals in BSFL frass on crops. Therefore, this study is intended to evaluate: (i) the migration and transformation of Cd during the pig manure–BSFL transformation process and (ii) the impacts of Cd on maize growth during the application of BSFL frass derived from pig manure containing different amounts of Cd.

## 2. Materials and Methods

### 2.1. Pig Manure Transformation by BSFL

Pig manure was taken from a pig farm in Jinghai District, Tianjin, China. The basic physical and chemical properties were as follows: pH 6.5, content of organic matter (OM) 89.0%, total nitrogen (TN) 1.9%, P_2_O_5_ 4.4%, K_2_O 2.0%, and Cd 0.14 mg kg^−1^. Four treatments were carried out by adding different concentrations of cadmium chloride (CdCl_2_) into pig manure as BSFL diet, namely CK (no Cd added), Cd-3 (3 mg kg^−1^ Cd added), Cd-15 (15 mg kg^−1^ Cd added), and Cd-30 (30 mg kg^−1^ Cd added). Each treatment was repeated 3 times and in total 12 tests were carried out.

The BSFL were taken from Tianjin Agricultural University (TJAU), Tianjin, China. For each test, 400 g of seven-day-old BSFL was cultured at 30 ± 2 °C in a plastic container (720 × 455 × 175 mm) and fed daily with pig manure (moisture content of about 65%). Feeding stopped after 8 days and the larvae were starved for 24 h to empty the digested manure. The BSFL and their frass were separated by manual sieving. The BSFL frass was naturally air-dried for further utilization.

### 2.2. Maize Growing Test

The soil for the planting test was collected from the West Campus of TJAU. The soil was air-dried, sieved, and fully mixed before use. The physicochemical properties of soil were as follows: pH 8.27, electrical conductivity (EC) 367 μs·cm^−1^, content of OM 23.85 g·kg^−1^, nitrate nitrogen (NO_3^−^_-N) 28.97 mg kg^−1^, available phosphorus 21.70 mg kg^−1^, and available potassium 235.55 mg kg^−1^. The maize variety used for the test was Zhengdan 958.

Four fertilization treatments (corresponding to 4 types of BSFL frass obtained, as mentioned in Section 2.1) were set up for the maize pot experiment, namely CK, Cd-3, Cd-15, and Cd-30, and one unfertilized experiment (without BSFL frass application) was conducted as CK0. Each treatment was repeated four times. For each replicate, a polyvinyl chloride (PVC) pot (23 cm in diameter, 18 cm in height) was filled with 6 kg of soil with the addition of 3% BSFL frass fertilizers. After watering thoroughly, the pots were kept in the shade for one week before planting. On 10 June 2019, the maize seeds were planted in the plastic pots after being soaked in water for 24 h, with 7 seeds per pot and routine management. After emergence, seedling thinning was carried out to keep 3 maize seedlings with similar growth in each pot. During the whole growth process of maize, the water content was maintained at about 60% of the field capacity.

The maize plants were harvested on 29 August 2019. The plant height was measured with a tape measure. The chlorophyll content and photosynthetic rate of leaves were measured using a chlorophyll meter (SPAD-502 Plus, Konica Minolta, Japan) and a photosynthetic rate meter (CI-340, CID, USA), respectively. When harvesting maize, the shoots (the aboveground part) were cut from the base of stem, and the roots (the underground part) were carefully taken out. The root was rinsed with deionized water and was dried by absorbing excess water. The aboveground and underground parts of the maize were oven-dried at 105 °C for 30 min, and then dried at 75 °C until they reached a constant weight. After recording the dry weight, different parts of the maize plants were crushed by a plant grinder, passed through a 0.25 mm nylon sieve, and then placed in plastic sealed bags for further analysis. After thoroughly mixing the soil samples in each pot, sampling was carried out using a quartering method. The soil samples were naturally dried by placing them in a cool and ventilated site. The soil samples were sieved with 1 mm and 0.25 mm sieves and then stored in plastic bags for further analysis.

### 2.3. Determination Methods

After the pig manure transformation test, the obtained BSFL were microwave-dried and their total weight was measured. The concentrations of Cd in the BSFL bodies and frass were determined as described previously [15]. Briefly, 0.5 g of the BSFL body sample was digested in a 10 mL mixture of HNO_3_ and H_2_O_2_ (4:1, *v/v*) by a CEM-MARS 6S microwave digestion system (CEM Corp, Matthews, NC, USA). For pig manure and BSFL frass, 0.5 g of crushed sample was digested in a mixture of 6 mL HNO_3_, 2 mL HCL, and 2 mL H_2_O_2_ by a CEM-MARS 6S microwave digestion system. The modified BCR method [15,18] was used to determine different Cd fractions in the pig manure and BSFL frass, including the weak acid-soluble fraction (F1), reducible fraction (F2), oxidizable fraction (F3), and residual fraction (F4). The total Cd concentration in both pig manure and BSFL frass was pre-treated in the same way as that of the residual fraction. All the Cd contents were determined by ICP-MS (iCAPQ, Thermo Scientific, Waltham, MA, USA).

The determination method of Cd in the maize plants was the same as that for pig manure. For the soil, 0.5 g of the sample was digested in a mixture of 6 mL HNO_3_, 2 mL HCl, and 2 mL HF by a microwave digestion system. The total Cd concentrations and four Cd speciations in the soil were determined using the same procedure as described for pig manure.

### 2.4. Data Statistical Analysis

Data were checked for normal distribution with the one-sample Kolmogorov–Smirnov test and for homogeneity of variances with Levene’s test. One-way analysis of variance (ANOVA) and the Duncan test were used to determine the differences among different groups of samples. When normal distribution and/or homoscedasticity were not achieved, data were subjected to the Kruskall–Wallis non-parametric test. Statistical analyses were performed using SPSS version 20.0 (SPSS Inc, Chicago, IL, USA) with a significance level of *p* < 0.05. Microsoft Excel 2019 and Origin version 2021 (OriginLab, Northampton, MA, USA) were used to generate additional plots.

## 3. Result and Discussion

### 3.1. Effects of Cd on Production Performance of BSFL

After an 8-day transformation period, 400 g of seven-day-old BSFL turned 18.5 kg of fresh pig manure (moisture content of 65%) into 2.59–2.73 kg of fresh larval biomass and 5.34–5.47 kg of dry larval frass (Table 1). The addition of Cd had no significant effect on the BSFL growth and the BSFL transformation performance of pig manure.

During the transformation process of multiple organic waste, BSFL usually exhibited strong tolerance to heavy metals [13,14,15]. Here, within the concentration range of Cd tested (3–30 mg kg^−1^), Cd in pig manure did not significantly influence the weight gain of BSFL, which concurred with the findings in a low Cd exposure (1.5–3 mg kg^−1^) during a BSFL–pig manure transformation test [19]. Wu et al. [17] found that exposures to Cd at a higher level (10–80 mg kg^−1^) did not significantly inhibit the weight gain of BSFL fed with wheat bran. Gao et al. [16] also reported that Cd (20–80 mg kg^−1^) had no effects on the survival and eclosion rates of BSFL. The metallothionein involved in a defense mechanism could elevate Cd storage capacity in insects [20]. Meanwhile, high levels of Cd could trigger the synthesis of HSP70 protein family in cells, which protected other proteins from the influence of Cd [21]. These reasons might explain the strong tolerance of BSFL to heavy metals.

### 3.2. Cd Contents in Pig Manure, BSFL Bodies, and BSFL Frass

**Figure 1:**
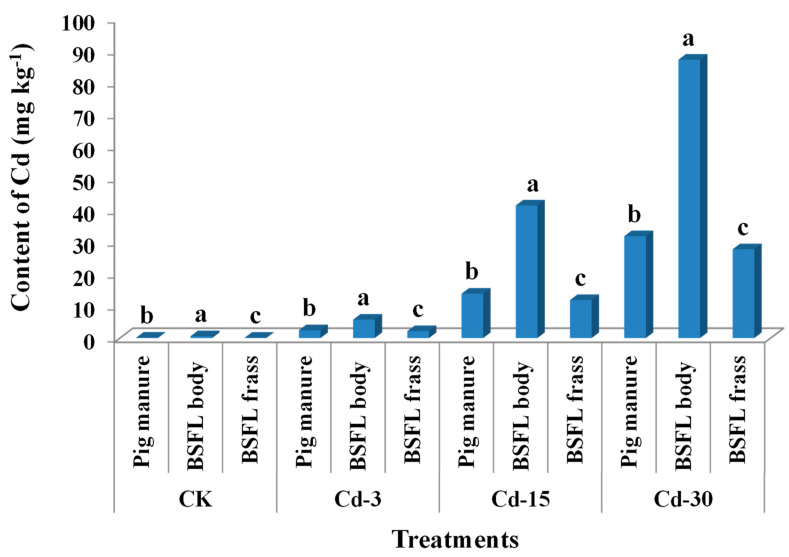
Contents of Cd (mean ± SE, *n* = 3) in pig manure, BSFL bodies, and BSFL frass under different treatments. Different letters denote significant difference at *p* < 0.05 under the same treatment.

The contents of Cd in pig manure under CK, Cd-3, Cd-15, and Cd-30 treatments were 0.14 mg kg^−1^, 2.46 mg kg^−1^, 13.91 mg kg^−1^, and 31.92 mg kg^−1^, respectively. After being transformed by BSFL, Cd from pig manure entered the BSFL bodies and frass. For all treatments, the Cd contents accumulated in BSFL bodies were significantly higher than those in BSFL frass and pig manure (Figure 1). In detail, Cd contents in larval bodies were 4.0-, 2.3-, 3.0-, and 2.7-times those of pig manure under CK, Cd-3, Cd-15, and Cd-30 treatments, indicating that Cd was highly accumulative in BSFL bodies. In turn, Cd contents in BSFL frass were remarkably lower than those in pig manure in all treatments (decreased by 10.9–19.8%).

The midgut of insects is the main site for the digestion and absorption of nutrients [22]. On the one hand, heavy metals in livestock manure can accumulate in the intestinal epithelial cells after entering the insect body. After the regeneration of the epithelial cells, metals along with old epithelial cells as intestinal metabolites are further excreted through insect frass [23]. On the other hand, heavy metals can also enter hemolymph through the basement membrane of midgut epithelial cells and bind with proteins, which are then retained in the insect body along with the transportation of hemolymph to other organs with secretion or storage functions [24].

Previous studies revealed that most of the heavy metals existing in feeding substrates could be excreted by BSFL frass [15,19]. However, due to the special absorption mechanism of Cd by BSFL, Cd seems to be more easily bioaccumulated in BSFL bodies as compared with other metals [14,21,25]. The current work also confirmed this concept. The concentrations of Cd accumulated in BSFL bodies were in a range of 0.6–87.2 mg kg^−1^, 2.3–4.0-times those in the respective pig manure fed to BSFL. This might bring a potential risk associated with Cd during the resource utilization of BSFL bodies. As a result of Cd accumulation in the larval bodies, the contents of Cd in BSFL frass were relatively decreased. Moreover, the contents of Cd in BSFL frass under CK and Cd-3 treatments were lower than the limit (3 mg kg^−1^) in China’s NY 525–2012 organic fertilizer standard, but those under Cd-15 and Cd-30 treatments were higher than the limit.

### 3.3. Effects of BSFL Frass Application on Maize Growth Parameters

The application of BSFL frass fertilizers promoted maize growth. Specifically, compared to the CK0 sample, the plant height, SPAD values, photosynthetic rate, and dry weight of the aboveground and underground parts of maize were significantly increased under BSFL frass application treatments (Table 2). The application of BSFL frass containing different Cd contents had no significant effects on maize plant height. However, Cd-30 treatment significantly reduced SPAD, photosynthetic rate, and dry weight of the aboveground and underground parts by 12.8%, 20.7%, 18.7%, and 16.1%, respectively, compared with CK. There were no significant differences between CK and other treatments (Cd-3 and Cd-15).

Studies have shown that BSFL frass fertilizers are rich in organic matter and multiple nutrients, and the rational application of larvae frass could enhance the growth of maize [26], rice [27], ryegrass [28], etc. In this study, BSFL frass application promoted maize growth, which further confirmed the great potential of larvae frass as organic fertilizers. Noticeably, the application of frass containing low or medium concentrations of Cd (Cd-3 and Cd-15) had no remarkable influence on maize growth, as only the 30 mg kg^−1^ Cd treatment significantly inhibited maize growth. This finding concurred with the results obtained by Zhao et al. [29]. Patra et al. [30] explained this phenomenon as the low metal concentration having a positive ‘stimulating effect’ on plants. However, when metal concentrations were too high in the environment, they had toxic effects on the plants’ growth and caused physiological and biochemical changes in seedlings. Bavi et al. [31] had similar findings on soybean seedling growth under Cd stress.

### 3.4. Cd Contents in Soil, and Aboveground and Underground Parts of Maize

**Figure 2:**
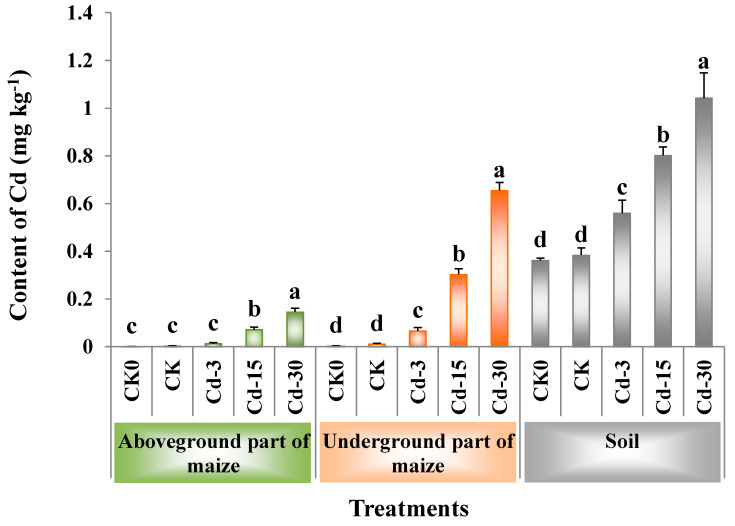
Contents of Cd (mean ± SE, *n* = 4) in soil, aboveground, and underground parts of maize under different treatments. Different letters denote significant difference at *p* < 0.05. CK0: Maize planting without any fertilizer application.

Figure 2 showed the contents of Cd in the soil, as well as the aboveground and underground parts of maize. When no exogenous Cd was added, the application of BSFL frass had no significant effects on the contents of Cd in the maize plants and soil (comparison between CK and CK0). With the increase in the amount of exogenous Cd added, the Cd contents in the aboveground and underground parts of maize increased by 3.3–47.0-times and by 4.9–57.6-times, respectively, compared with the CK sample, while those in the soil increased by 0.5–1.7-times. Among all the treatments, Cd-30 had the highest contents of Cd in the aboveground (0.15 mg kg^−1^) and underground (0.66 mg kg^−1^) parts of maize, as well as in the soil (1.04 mg kg^−1^).

For maize planting, the application of BSFL frass containing different contents of Cd significantly increased the contents of Cd in the soil. Among them, the Cd contents in soil under Cd-15 and Cd-30 treatments exceeded the standard for soil contamination of agricultural land in China GB15618-2018 (pH > 7.5, Cd ≤ 0.6 mg kg^−1^). BSFL frass application also significantly increased the Cd contents in the aboveground and underground parts of maize, indicating that Cd in BSFL frass could migrate from fertilized soil to maize plants and accumulate in the plants. Wu et al. [32] also found that the long-term use of organic fertilizers containing heavy metals increased the Cd contents in both the soil and maize, and Cd contents in the maize stalks were significantly and positively correlated with those in the soil. Additionally, Cd contents in the underground part of maize were higher than those in the aboveground part, which was consistent with the previous research results [33].

### 3.5. Speciation of Cd in Pig Manure, BSFL Frass, and Soil

**Figure 3:**
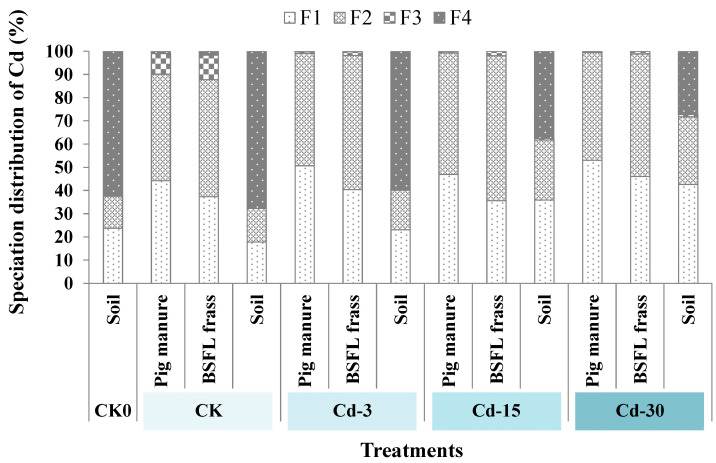
The changes in Cd speciation in soil, pig manure, and BSFL frass under different treatments. F1: weak acid-soluble fraction; F2: reducible fraction; F3: oxidizable fraction; F4: residual fraction.

Figure 3 revealed the speciation distribution of Cd in pig manure, BSFL frass, and soil. Cd mainly existed in the forms of F1 and F2 (total percentage of 90.1–99.5%) in pig manure and BSFL frass. For pig manure, the addition of exogenous Cd increased the proportions of F1-state Cd. In detail, the proportions of F1-state Cd in pig manure under Cd-3, Cd-15, and Cd-30 treatments increased to 50.7%, 46.9%, and 53.0%, respectively, compared with CK (44.2%). After the transformation by BSFL, the proportions of F1-state Cd in BSFL frass declined (13.1–24.1%) compared with raw pig manure. For soil, the application of BSFL frass (derived from CK pig manure) decreased the proportion of F1-state Cd compared with the CK0 sample. However, with the increase in exogenous Cd added, BSFL frass application increased the fractions of F1-state Cd from 17.8% in CK soil to 23.1%, 35.9%, and 42.5%, respectively, in Cd-3, Cd-15, and Cd-30 soil. Meanwhile, BSFL frass application reduced the proportions of F4-state Cd in the soil (which decreased from 67.7% in CK soil to 27.2–59.9% in soil under Cd-added treatments).

The influence of heavy metals in organic fertilizers on crop growth is not only related to the total amount of heavy metals, but also to the form of heavy metals. Metal speciation directly affected the toxicity, migration, and circulation of heavy metals in nature [34]. Among the four types of metal speciation, the weak-acid-dissolved (F1) state is most easily absorbed by crops and has high biological activity, while the residual (F4) state has the lowest biological activity. The results of this work showed that F1 proportions in the pig manure with and without exogenous Cd addition were relatively higher (44.2–53.0%). After BSFL transformation, the fractions of F1-state Cd in BSFL frass decreased (37.3–46.0%) compared to pig manure. After BSFL frass was applied to the soil, the proportions of F1-state Cd were further lowered (17.8–42.5%). Conversely, the fractions of F4-state Cd in pig manure and BSFL frass were low (0.007–1.7%) and those significantly increased (27.2–67.7%) after BSFL frass application into soil. This suggested that BSFL transformation could reduce the harm of Cd sourced from pig manure. In addition, after BSFL frass was applied to the soil as organic fertilizer, the soil could fix heavy metals sourced from BSFL frass fertilizer and reduce the biological activities of heavy metals, as well as the metal damage to crops. Similar findings were also observed in other research. For example, Tian et al. [35] found that the bioavailable fraction of Cd was 41–60% of the total amount of exogenous Cd entering the soil after seven days. With the extension of time, the bioavailable fraction of Cd further decreased. Previous studies [36,37,38] also showed that soil can immobilize exogenous heavy metals.

### 3.6. Migration of Cd in Pig Manure–BSFL Frass–Soil–Maize System

**Figure 4:**
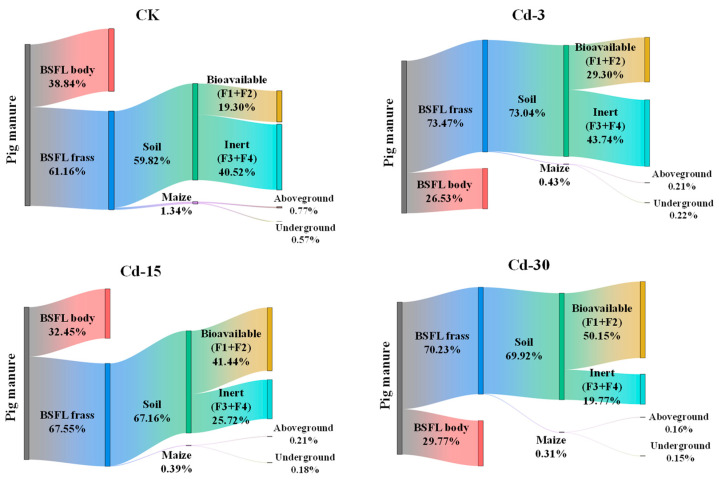
Migration and distribution of Cd in pig manure–BSFL frass–soil–maize system under different treatments.

After being transformed by BSFL, Cd in pig manure was partially transferred into BSFL frass (61.16–73.47%) under different treatments and the rest was transferred into BSFL bodies (26.53–38.84%) (Figure 4). This process, to some extent, reduced the environmental risk of pig-manure-sourced Cd entering the soil and crops, but at the same time, increased the potential risk associated with Cd when using larvae as animal feed ingredients.

After BSFL frass was applied to the soil as an organic fertilizer for maize planting, a small part (0.31–1.34%) of Cd was absorbed by the maize, while most of the Cd remained in the soil (59.82–73.04%). For Cd remaining in the soil, the bioavailable fraction with higher biological activity (F1 + F2) accounted for 19.03–50.15%, while the inert fraction with lower biological activity (F3 + F4) accounted for 19.77–40.52%. With the increase in exogenous Cd concentration in pig manure, the bioavailable fractions of Cd in the soil increased, while the inert fractions reduced. Compared with CK, the bioavailable fraction of Cd in the soil under Cd-30 treatment increased from 19.30% to 50.15%, and the inert fraction decreased from 40.52% to 19.77%. In addition, the proportions of Cd entering the aboveground part of the maize plant were 0.16–0.77%, and those entering the underground part were 0.15–0.57%. With the increase in exogenous Cd concentration, the proportions of Cd in both the aboveground and underground parts of maize decreased.

## 4. Conclusions

The addition of different concentrations of Cd to pig manure did not have significant effects on the BSFL growth and the BSFL transformation performance of pig manure. After BSFL transformation, 61.2–73.5% of Cd in pig manure entered the BSFL frass, and 26.5–38.8% of Cd accumulated in the BSFL bodies. During maize planting, the application of BSFL frass fertilizers originating from pig manure containing different Cd amounts increased the Cd contents in soil and maize plants, but only BSFL frass under a high level of Cd treatment (30 mg kg^−1^) significantly inhibited maize growth. Only a small part (0.31–1.34%) of Cd in pig manure finally entered the maize plants, and most Cd remained in the soil. During the Cd migration process of pig manure–BSFL frass–soil, the proportion of Cd in the bioavailable state decreased, while that in the residual state increased.

## Figures and Tables

**Table 1:** Production performance of transforming pig manure by BSFL.

Treatment	Fresh Weight of Obtained BSFL (kg)	Dry Weight of Obtained BSFL (kg)	Dry Weight of BSFL Frass (kg)
CK	2.59 ± 0.08a	0.70 ± 0.016a	5.47 ± 0.26a
Cd-3	2.71 ± 0.06a	0.73 ± 0.022a	5.34 ± 0.13a
Cd-15	2.73 ± 0.08a	0.74 ± 0.022a	5.35 ± 0.12a
Cd-30	2.69 ± 0.06a	0.73 ± 0.034a	5.37 ± 0.13a

Note: Different letters in the same column indicate significant differences between treatments at *p* < 0.05 level; mean ± SE, *n* = 3.

**Table 2:** Effects of different BSFL frass application on maize growth parameters.

Treatment	Plant Height (cm)	Chlorophyll (SPAD)	Photosynthetic Rate (μmol/(m^2^·s))	Dry Weight of Aboveground Parts (g)	Dry Weight of Aboveground Parts (g)
CK0	103.6 ± 3.4b	15.6 ± 0.5c	17.5 ± 2.1c	34.8 ± 2.2c	6.5 ± 0.9c
CK	148.8 ± 5.3a	25.7 ± 1.6a	30.5 ± 3.8a	94.3 ± 7.5a	19.3 ± 1.5a
Cd-3	146.6 ± 5.8a	25.3 ± 1.6a	30.8 ± 2.4a	93.6 ± 8.9a	19.0 ± 1.0a
Cd-15	150.5 ± 2.0a	25.4 ± 1.3a	30.7 ± 2.1a	93.9 ± 5.0a	18.9 ± 1.4a
Cd-30	143.3 ± 5.2a	22.4 ± 0.7b	24.2 ± 3.0b	76.7 ± 8.6b	16.2 ± 1.3b

Note: Different letters in the same column indicated significant differences between treatments at *p* < 0.05 level; mean ± SE, *n* = 4.

## Data Availability

The datasets used and/or analyzed during the current study are available from the corresponding author on reasonable request.
